# Conveyance of sofosbuvir through vesicular lipid nanocarriers as an effective strategy for management of viral meningitis

**DOI:** 10.1039/d3ra06540e

**Published:** 2023-11-15

**Authors:** Bhabani Sankar Satapathy, Pralaya Kumar Sahoo, Snigdha Pattnaik, Amit Kumar Nayak, Laxmidhar Maharana, Rudra Narayan Sahoo

**Affiliations:** a School of Pharmaceutical Sciences, Siksha ‘O’ Anusandhan (Deemed to be University) Kalinga Nagar Bhubaneswar Odisha 751003 India snigdhapattnaik@soa.ac.in

## Abstract

This study aimed to deliver a potential water-soluble antiviral drug (sofosbuvir) through optimized vesicular lipid nanocarriers (LNs) to the rat brain as a novel strategy against viral meningitis. A 2^3^ factorial design approach was established to assess the effect of formulation composition and process variables on the physicochemical properties of the LNs. Sofosbuvir-loaded LNs (SLNs) were developed by lipid layer hydration method utilizing optimized parameters and evaluated for various *in vitro* characterizations like FTIR, DSC, XRD, FESEM, vesicle size, zeta potential, drug carrying capacity and drug release. Plasma and brain pharmacokinetic (PK) studies were conducted in Sprague-Dawley rats. FTIR data depicted the absence of any major interaction between the drug and the excipients. DSC revealed a sharp endothermic peak for the drug. XRD showed the amorphic nature of the SLNs. Optimized SLNs were spherical as depicted from FESEM with 42.43 nm size, −49.21 mV zeta potential, 8.31% drug loading and sustained drug release *in vitro*. Plasma/brain PK studies depicted significant improvement in key PK parameters, *viz*. AUC, AUMC, MRT, and *V*_d_, compared to those for the free drug. A more than 3.5-fold increase in MRT was observed for optimized SLNs (11.2 h) in brain tissue compared to the free drug (3.7 h). *Ex vivo* hemolysis data confirmed the non-toxic nature of the SLNs to human red blood cells. *In silico* docking study further confirmed strong interaction between the drug and selected protein 4YXP (herpes simplex) with docking score of −7.5 and 7EWQ protein (mumps virus) with docking score of −7.3. The optimized SLNs may be taken for further *in vivo* studies to pave the way towards clinical translation.

## Abstract 2

This study aimed to deliver a potential water-soluble antiviral drug (sofosbuvir) through optimized vesicular lipid nanocarriers (LNs) to the rat brain as a novel strategy against viral meningitis.

## Graphical abstract

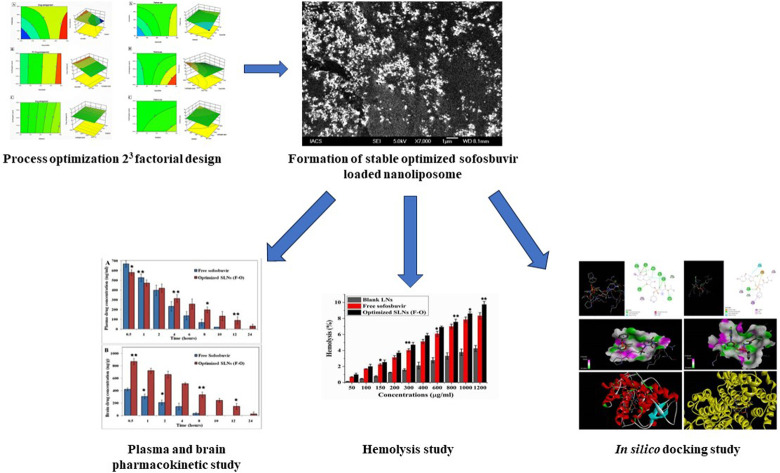

## 1. Introduction

Delivery of therapeutic agents to the brain is a perplexing task due to the presence of the blood–brain barrier (BBB).^1^ Especially, delivery of water-soluble drugs has been most challenging owing to the highly lipophilic nature of the BBB.^2^ The obstructions imposed by the BBB indeed make the successful treatment of many brain-related diseases difficult, including meningitis.^3^ However, recent advancements in drug delivery technology have provided some hope of overcoming this challenge. In this context, exploration of nanolipoidal carriers, such as nanoliposomes, solid–lipid nanoparticles, niosomes, and lipid nanoconstructs, has been in the limelight over the past decade to improve the brain delivery of conventional drugs.^4^ Among various novel carriers, vesicular lipid nanocarriers, commonly referred to as nanoliposomes (LNs), have become a popular strategy for effective delivery of hydrophilic drugs across the BBB to treat brain diseases.^5^

LNs are nano-sized self-assembled vesicles that are constructed from cholesterol and natural phospholipids, enclosing a small aqueous core. In view of their unique architecture, LNs can successfully transport both hydrophilic and hydrophobic entities in their structural scaffold.^6^ Among various nanocarriers, LNs enjoy special status in the drug delivery arena for their biocompatibility and cell-mimicking nature. Due to their nano-size with high lipophilic nature, LNs are considered potential novel carriers for the delivery of hydrophilic therapeutics to the brain.^7^ Differences in lipid composition, size, surface charge, and preparation method give LNs with a wide range of properties. Furthermore, the “fluidity” or rigidity of the bilayer and its charge are determined by the choice of bilayer components.^8^ LNs can engulf hydrophilic/hydrophobic compounds, keep them from disintegrating into one another, and release them at predetermined targets in a sustained manner.^9^

Sofosbuvir is an established antiviral drug recommended for hepatitis C and other viral diseases.^10^ However, being a BCS class III drug (low permeability/high solubility), its delivery across the BBB is a big challenge. It has water solubility of 105 mg L^−1^ at 25 °C. Although the delivery of sofosbuvir through nanocarrier-based platforms has been attempted in some recent investigations, its brain delivery has not been reported so far. In 2022, El-Shafai *et al*. reported delivery of sofosbuvir through β-cyclodextrin-modified chitosan nanoparticles for treating hepatitis C. Improved drug loading, drug release and cytotoxic activity were observed for sofosbuvir-loaded experimental nanoparticles.^11^ Another recent work reported cytotoxic testing of sofosbuvir-modified dextran-stabilized silver nanoparticles (AgNP) on the Huh-7 cell line. Data depicted lower cytotoxicity of the sofosbuvir-loaded AgNP on the tested liver cell line than that of the free drug.^12^ Moreover, sofosbuvir has been reported to protect human brain organoids against COVID-19 infection.^13^ This study showed that sofosbuvir could effectively protect from neuronal damage and impaired synaptogenesis in SARS-CoV-2 infection, which provides another rationale for use of sofosbuvir in brain disorders.

The aim of the current work was to investigate the potential of optimized LNs to successfully deliver sofosbuvir to the rat brain as a strategy to treat viral meningitis. Sofosbuvir-loaded LNs (SLNs) were developed using a 2^3^ factorial design approach to find the optimum formulation. Optimized SLNs were evaluated for various physicochemical characteristics. Plasma and brain pharmacokinetic (PK) studies were conducted in Sprague-Dawley rats to estimate the drug concentration in the brain and plasma. We also wanted to see the *in silico* interaction of some crucial meningitis-related virus protein(s) with sofosbuvir. Considering the involvement of herpes simplex and mumps viruses in majority of brain meningitis cases, selected proteins of these two viruses were docked with the drug to establish its rationality for use in meningitis. To the best of our knowledge, encapsulation of sofosbuvir through optimized LNs and its availability in brain tissue is yet to be investigated. Formulation optimization through factorial design, *in vitro* evaluation, *in vivo* plasma/brain PK with *ex vivo* hemolysis data adds uniqueness to the work, which provides necessary insights for furthering research on clinical translation of SLNs to treat viral meningitis.

## 2. Experimental

### 2.1 Materials

Cipla Ltd Goa, India provided the drug sofosbuvir as a gift sample. Soy-α-lecithin (SL; Hi-Media Laboratories Pvt. Ltd, Mumbai, India), cholesterol (CHL; E Merck Ltd, Mumbai, India), and butylated hydroxyl toluene (BHT; SRL Chemicals, Mumbai, India) were used. All the other chemicals/reagents used for the study were of analytical grade.

#### 2.1.1 Animals

For PK studies, healthy Sprague-Dawley rats (both sexes) weighing 90–120 g were used. All animal-related experiments were conducted after getting the necessary approval from the Animal Ethical Committee of Siksha ‘O’ Anusandhan University, Bhubaneswar, Odisha. Before starting the experiments, animals were acclimatized for 14 days in the institute's animal house. Animals were kept in polypropylene cages and were fed with standard diet and drinking water *ad libitum*. The temperature (22 °C) and humidity (55%) of the animal house were maintained properly with a 12 h light/dark cycle.

### 2.2 Method of development of experimental SLNs

Experimental SLNs were prepared as per a previously reported method with modifications when required.^14,15^ Briefly, SL and CHL at the optimized concentrations were taken in 10 ml of chloroform and vigorously shaken. To prevent oxidation, BHT (1% w/v) was also added to the dispersion. With the help of a rotary vacuum evaporator (Aditya Scientific, Mumbai, India), chloroform was removed from the dispersion, leaving a thin lipid layer inside the round-bottom flask. The flask was kept in a desiccator overnight for complete drying of the film. On the second day, 50 ml of phosphate buffer (pH 7.4) was added to the lipid film layer for hydration with the help of a rotary evaporator for 1 h. The dispersion was then sonicated (bath sonicator, Sonix, Vibracell) for 20 min to obtain small unilamellar vesicles. The formulation was stored in the refrigerator followed by centrifugation using a cold centrifuge (Remi centrifuge, R 8C plus, India) at 17 000 rpm for 30 min. The collected pellets were stored in the refrigerator for 4 h and lyophilized to dry powder form using a lyophilizer (C-GEN BIOTECH -80 Deg laboratory freeze dryer, Pune, India).

### 2.3 Process optimization and *in vitro* characterization of experimental SLNs loaded with sofosbuvir

#### 2.3.1 Experimental design

A 2^3^-factorial design (three-factor and two-level) was employed for SLN formulation optimization. Amount of SL (X_1_), amount of CHL (X_2_) and centrifugation speed (X_3_), as the prime selected three factors (independent variables), were varied at two levels: low (−) and high (+). The drug entrapment efficiency (DEE), and particle size (nm) were analysed as the two responses (dependent variable). Design-Expert 8.0.6.1 software (Stat-Ease Inc., USA) was used for generation, evaluation and analysis of the statistical experimental design (2^3^-factorial design) for SLN formulation optimization.

#### 2.3.2 Fourier transform-infrared spectroscopy (FTIR) analysis

To check for interactions between the drug and selected lipid/excipients, FTIR analysis was conducted as per the previously reported method.^16^ The selected materials (pure drug, SL, CHL, BHT, physical mixture, SLNs and blank LNs) were analysed in an FTIR-ATR analyser (Magna-IR 750, series-II, Nicolet instruments, Madison, Wisconsin, USA) over the wavenumber range of 600–4000 cm^−1^.

#### 2.3.3 Differential scanning calorimetry (DSC)

DSC is a thermal analysis technique used to determine the amount of heat flowing into or out of a sample as a function of temperature or time. DSC analysis was carried out for the pure drug, SLNs and blank LNs (without drug) with a heating rate of 10 °C per min across the temperature range of 30 °C to 280 °C using a differential scanning calorimeter (Mettler Toledo DSC 1, Switzerland).^17^

#### 2.3.4 X-ray diffraction analysis (XRD)

XRD is an analytical tool that can be used to study a material's crystallographic structure.^18^ XRD patterns of pure sofosbuvir, blank LNs (without drug) and SLNs were analysed using an X-ray diffractometer (Model: UItima IV) with nickel-filtered Cu Kα radiation (=1.5406 A). The voltage and current ranged from 20 to 60 kV and 2 to 60 mA, respectively. At a scan speed of 1° min^−1^, measurements were taken in the angular scan range of 5° to 40° (2*θ*).

#### 2.3.5 Percentage yield determination

The percentage yield is a measure of production efficiency that is derived by comparing the actual yield of experimental SLNs to the theoretical yield using stoichiometric calculations.^14^ The following formula was used to calculate the yield (%) of SLNs.Yield% = (actual yield ÷ theoretical yield) × 100

#### 2.3.6 Estimation of drug loading (%) and drug loading efficiency (%)

A measured amount of the SLNs was mixed with water : ethanol (6 : 4) and vortexed for 30 min. The dispersion was then centrifuged at 17 000 rpm for 30 min.^14,19^ The supernatant was collected and the absorbance was measured at 280 nm using a UV-visible spectrophotometer (Beckman, Fullerton, CA, USA). The following formula was used to calculate the drug loading (%) and drug loading efficiency (%):Drug loading (%) = (amount of drug in LNs/amount of LNs obtained) × 100Drug loading efficiency (%) = (practical drug loading/theoretical drug loading) × 100

#### 2.3.7 Evaluation of average vesicle size, polydispersity index (PDI) and zeta potential

The size distribution of colloidal nanocarriers can be determined using techniques such as dynamic light scattering (DLS), which measures the intensity of scattered light by particles in a colloidal dispersed.^20^ Zeta potential, on the other hand, is a measure of the electrical charge on the surface of particles in a colloidal dispersion. The average vesicle diameter (Z-average), polydispersity index (PDI), and zeta potential of the experimental SLNs were measured using a DLS instrument (DLS nano ZS, Zetasizer, Malvern Instrument Ltd, Malvern, UK).

#### 2.3.8 Field emission scanning electron microscopy (FESEM)

An electron microscope (JSM 6100; JEOL, Tokyo, Japan) was used to examine the surface morphology of the experimental SLNs.^21^ Lyophilized SLNs were first dispersed over carbon tape. Gold coating was applied over the powdered samples at a voltage of 10 kV (for 5 min). Finally, the samples were analysed using FESEM under liquid nitrogen conditions.

#### 2.3.9 Determination of drug release *in vitro* (dialysis method)

To estimate the amount of drug release from the experimental SLNs, the dialysis method was employed.^22^ A dialysis bag (Hi Media dialysis membrane-60, Mumbai, India) was soaked in phosphate-buffered saline (PBS, pH 7.4) overnight. The dialysis bag used for this work is partially permeable and has a molecular weight cut off between 12 000 to 14 000 Da. The pore size of the dialysis bag was 2.4 nm, which is generally considered ideal for osmosis-related release studies. Lyophilized SLNs (10 mg) were dispersed in PBS and transferred into a dialysis bag. Both sides of the dialysis bag were closed with thread and then it was hung on a stand in a beaker containing 50 ml of PBS (release medium). The whole setup was placed on a magnetic stirrer and stirred at 300 rpm with a magnetic bead. At predetermined time intervals (0.25 h, 0.5 h, 1 h, 2 h, 3 h, 4 h, 5 h, 6 h, 7 h, 8 h, 12 h, and 24 h), samples (3 ml) were withdrawn from the beaker and replaced with fresh release medium. The samples were analysed by UV-visible spectrophotometer at 280 nm, using PBS as the blank. From the standard calibration curve, the concentration of sofosbuvir in the sample was calculated.

#### 2.3.10 Drug release kinetics estimation

*In vitro* drug release kinetics study is used to evaluate the release mechanism of drug from a dosage form. The drug release profile was analysed using various mathematical models, such as zero-order (cumulative amount of drug released *vs.* time), first-order (logarithmic value of cumulative amount of drug released *vs.* time), Higuchi models (cumulative amount of drug released *vs.* square root of time), Korsmeyer–Peppas (logarithmic value of cumulative amount of drug released *vs.* logarithmic value of time), and Hixson–Crowell (cube root of drug percentage to be released *vs.* time).^23^ The corresponding *R*^2^ values were determined.

### 2.4 *In vivo* studies

#### 2.4.1 *In vivo* brain and plasma PK studies

The PK profile of sofosbuvir was investigated *in vivo* in both plasma and brain tissue in healthy Sprague-Dawley rats. For the study, the animals were divided into three treatment groups *viz.*, (Group 1) Free sofosbuvir-treated, (Group 2) Optimized SLNs-treated group, and (Group 3): Control (saline treated). The animals were injected with 10 mg kg^−1^ free sofosbuvir and optimized SLNs (containing an equivalent amount of sofosbuvir) intravenously *via* the tail vein. At specific time intervals (0.5 h, 1 h, 2 h, 6 h, 8 h, 10 h, 12 h, and 24 h) animals were sacrificed. Blood samples were collected through heart puncture into pre-heparinized tubes and immediately centrifuged at 1000 rpm for 5–10 min to collect plasma. For determination of brain PK, the brains of the animals were isolated at pre-fixed time intervals as mentioned above. Brains were crushed using a tissue homogenizer and centrifuged at 5000 rpm for 30 min to obtain the supernatant. Drug was extracted from the supernatant by the reported method and the concentration of drug in the final sample was analysed using LC-MS/MS.^22^

#### 2.4.2 LC-MS/MS condition

LC-MS/MS was utilized to estimate the concentration of sofosbuvir present in the blood/brain post *i.v.* administration of optimized SLNs or pure sofosbuvir in selected animal groups. Briefly, 100 μl of plasma/brain sample was precipitated with acetonitrile (300 μl) and vortexed for 10 min. Samples were then centrifuged at 4000 rpm (10 min). About 100 μl of sofosbuvir-loaded supernatant was diluted with 100 μl of deionized water. The sample was then dried under an inert atmosphere then reconstituted with mobile phase (acetonitrile : water, 20 : 80 v/v).^23^ Internal standard (quinine sulfate) solution (50 μl) was added to each sample. About 20 μl of sample was finally injected into the LC-MS/MS system (Agilent 6410, Triple Quad MS-MS, Agilent, USA). Important pharmacokinetic parameters *viz*. area under the curve (AUC), mean residence time (MRT), area under the first moment curve (AUMC), volume of distribution (*V*_d_), and total clearance (Cl) were determined using the non-compartmental model in Phoenix WinNonlin software (Version 6.0, Pharsight Corporation; Cary, NC).

#### 2.4.3 *Ex vivo* hemolysis study

An *ex vivo* hemolysis assay was performed to assess the biocompatibility and safety profile of the optimized SLNs/blank LNs/free sofosbuvir as per the previously reported method.^24^ Blood samples were taken from the Sprague-Dawley rats. The samples were collected in pre-heparinized tubes, and then cold centrifuged at 5000 rpm for 5–7 minutes. The red blood cells (RBCs) were then washed in PBS (pH 7.4). A measured amount of RBC suspension (190 μl) was placed in a 96-well plate and treated with varied concentrations of optimized SLNs, free sofosbuvir, and blank LNs. Double distilled water was taken as the positive control. The samples were incubated for 1 h at 37 °C and centrifuged for at 5000 rpm (5 min) to separate the un-lysed RBCs. The supernatant was then collected, and the corresponding absorbance was measured at 280 nm.

### 2.5 *In silico* docking study

The PubChem website was used to obtain the 2D structure of sofosbuvir. The crystal structures of target viral proteins were obtained from the Protein Data Bank (http://www1.rcsb.org/). The molecular docking technique comprised dehydration of all proteins, separation and storage of original ligands, and molecular docking was performed using AutoDockTools-1.5.6.^25^ PyRx software was used to visualize the components of protein molecular docking.

### 2.6 Statistical analysis

To ensure accuracy and reproducibility, all tests were performed in triplicate. The results are expressed using the average and standard deviation (SD). One-way analysis of variance (ANOVA) was utilized to examine statistical data using the Origin2023b program, followed by Tukey's *post hoc* test. Differences were considered statistically significant when *p* < 0.05 was used with 95% confidence.

## 3. Results

### 3.1 Formulation optimization and *in vitro* studies

#### 3.1.1 Formulation optimization by 2^3^ factorial design

For the 2^3^ factorial design, a total eight trial formulations SLNs (sofosbuvir-loaded) were proposed by Design-Expert 8.0.6.1 software (Stat-Ease Inc., USA) for three factors (independent variables), namely amount of SL (X_1_), amount of CHL (X_2_) and centrifugation speed (X_3_), which were varied at two different levels (high and low). The effects of these independent variables on DEE (%), and particle size (nm) were investigated as optimization response parameters in the present study. An overview of the experimental trial and the observed responses is presented in Table 1.

**Table 1:** 2^3^ factorial design for formulation optimization of sofosbuvir-loaded vesicular lipid nanocarriers (SLNs) and their observed response values along with coded values in brackets^a^

Code	Soya lecithin (mg) X_1_	Cholesterol (mg) X_2_	Centrifugation speed (rpm) X_3_	Responses	
				DEE (%)	Particle size (nm)
F-1	400 (+1)	250 (+1)	17 000 (+1)	68.22	60.12
F-2	400 (+1)	250 (+1)	10 000 (−1)	70.12	79.32
F-3	400 (+1)	75 (−1)	10 000 (−1)	74.32	82.45
F-4	100 (−1)	75 (−1)	10 000 (−1)	49.75	45.12
F-5	400 (+1)	75 (−1)	17 000 (+1)	74.32	68.22
F-6	100 (−1)	250 (+1)	17 000 (+1)	64.79	67.12
F-7	100 (−1)	75 (−1)	17 000 (+1)	49.75	54.31
F-8	100 (−1)	250 (+1)	10 000 (−1)	64.59	65.21
F-O	100	65	10 000	Actual values	
				51.55	42.43
				Predicted values	
				48.63	44.29
		Error%		4.81	4.20

a Mean ± S.D.; *n* = 3.

The Design-Expert 8.0.6.1 software provided suitable polynomial model equations involving individual main factors and interaction factors. The model equation relating DEE (%) as response became:DEE (%) = +30.03 + 0.12 X_1_ + 0.13 X_2_ + 1.71 × 10^−4^ X_3_ − 3.81 × 10^−4^ X_1_X_2_ − 4.52 × 10^−7^ X_1_X_3_ − 7.76 × 10^−7^ X_2_X_3_ [*R*^2^ = 0.9993; *F*-value = 248.56; *p* < 0.05]

The model equation relating vesicle size (nm) as response became:Vesicle size (nm) = −5.01 + 0.26 X_1_ + 0.20 X_2_ + 2.67 × 10^−3^ X_3_ − 4.20 × 10^−4^ X_1_X_2_ − 1.06 × 10^−5^ X_1_X_3_ − 5.00 × 10^−6^ X_2_X_3_ [*R*^2^ = 0.9994; *F*-value = 264.03; *p* < 0.05]

The results of ANOVA, as shown in Table 2, indicate that all these models (DEE, %, and vesicle size, nm) are significant (*p* < 0.05) for all response parameters investigated. Model simplification was carried out by eliminating non-significant terms (*p* > 0.05) in polynomial equations, giving:DEE (%) = +30.03 + 0.12 X_1_ − 3.81 × 10^−4^ X_1_X_2_Vesicle size (nm) = −5.01 + 0.26 X_1_ − 4.20 × 10^−4^ X_1_X_2_ − 1.06 × 10^−5^ X_1_X_3_

**Table 2:** Summary of ANOVA for response parameters

Source	Sum of square	d.f.^a^	Mean square	*F*-Value	*p*-Value (prob > *F*)
**DEE (%)**					
Model	672.96	6	112.16	248.56	0.0485
X_1_	424.86	1	424.86	941.52	0.0207
X_2_	46.95	1	46.95	104.04	0.0622
X_3_	0.45	1	0.45	1.00	0.5000
X_1_X_2_	199.80	1	199.80	442.77	0.0302
X_1_X_3_	0.45	1	0.45	1.00	0.5000
X_2_X_3_	0.45	1	0.45	1.00	0.5000

**Vesicle size (nm)**					
Model	1056.67	6	176.11	264.03	0.0471
X_1_	425.59	1	425.59	638.05	0.0252
X_2_	58.70	1	58.70	88.00	0.0676
X_3_	62.33	1	62.33	93.44	0.0656
X_1_X_2_	243.43	1	243.43	364.96	0.0333
X_1_X_3_	247.87	1	247.87	371.60	0.0330
X_2_X_3_	18.76	1	18.76	28.12	0.1187

a d.f. = degrees of freedom.

**Fig. 1:**
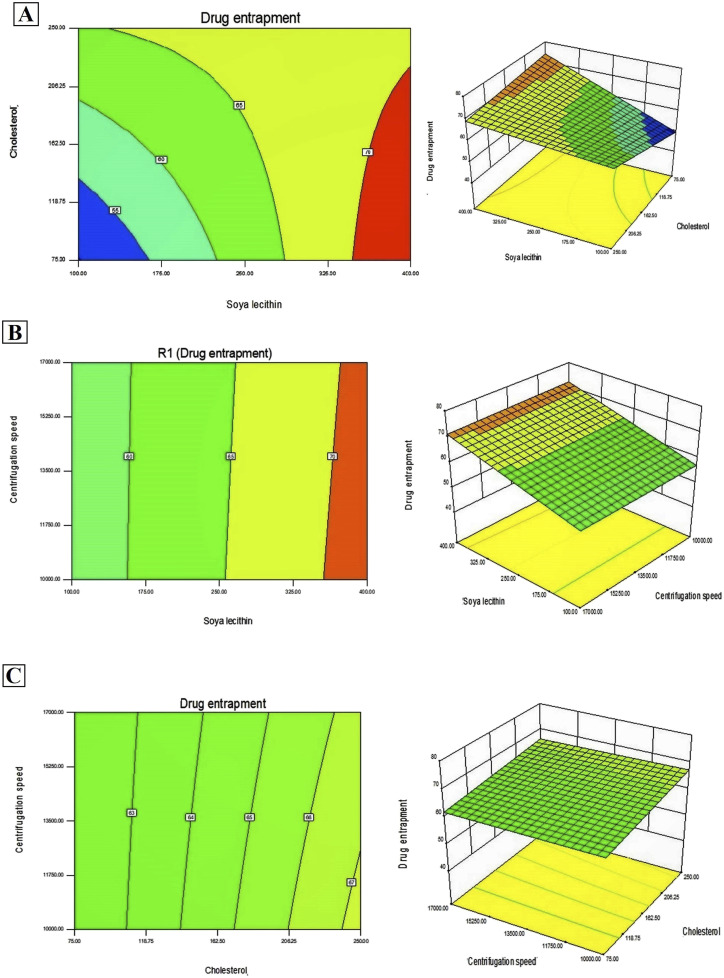
Three-dimensional response surface plots and corresponding two-dimensional contour plots relating effects of various independent variables: (A) effect of SL (X_1_) and CHL (X_2_), (B) effect of SL (X_1_) and centrifugation speed (X_3_), (C) effect of CHL (X_2_) and centrifugation speed (X_3_) on the measured response of drug encapsulation efficiency percentage (DEE %).

**Fig. 2:**
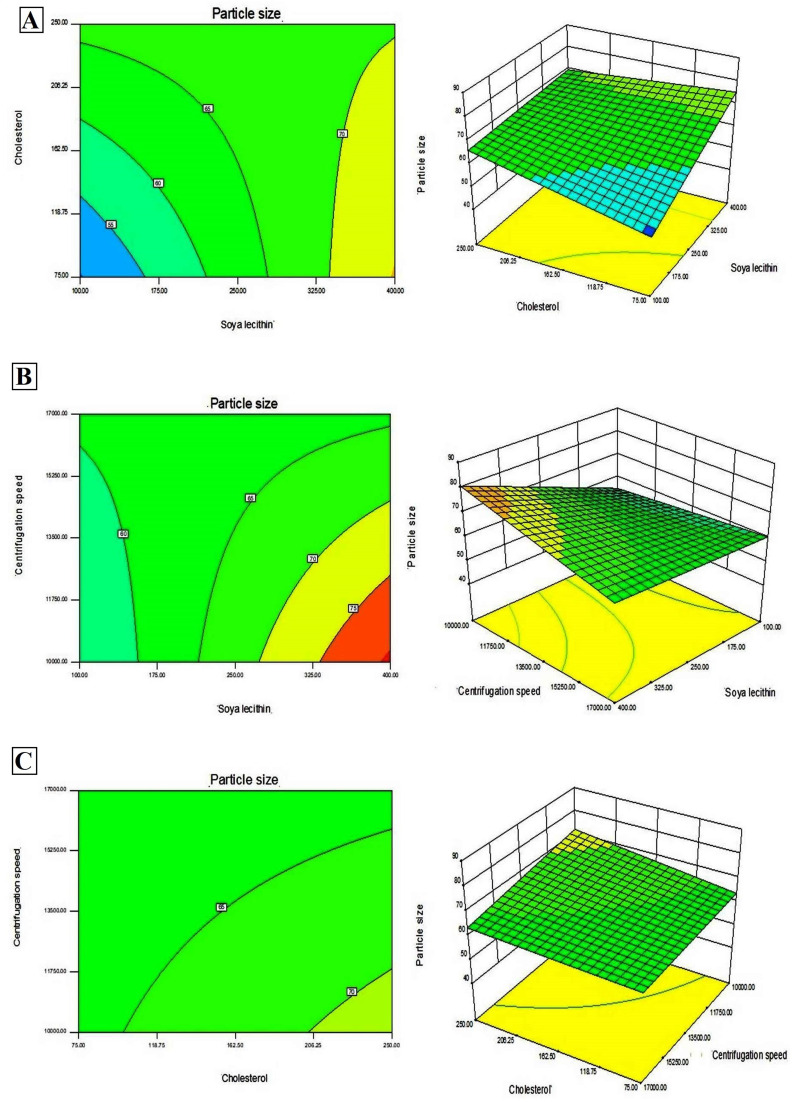
Three-dimensional response surface plots and corresponding two-dimensional contour plots relating effects of various independent variables: (A) effect of SL (X_1_) and CHL (X_2_), (B) effect of SL (X_1_) and centrifugation speed (X_3_), (C) effect of CHL (X_2_) and centrifugation speed (X_3_) on the measured response of vesicle size (nm).

In addition, Design-Expert 8.0.6.1 software generated three-dimensional response surface plots and corresponding two-dimensional contour plots relating various measured responses. These plots were presented to estimate the effects of the independent variables on each response (Fig. 1 and 2). A numerical optimization technique using the desirability approach was employed to develop an optimized formulation (F-O) with the desired response. The desirable ranges of independent variables were restricted to: X_1_ = 100 mg, X_2_ = 60 mg and X_3_ = 10 000; whereas the desirable ranges of responses were restricted to target: DEE → 70%, and vesicle size → 45 nm. In order to evaluate the optimization capability of models generated according to the results of 2^3^ factorial design, the optimized formulation (F-O SLNs) was prepared using the optimal process variable settings. The optimized SLNs (F-O) were evaluated for DEE (%), and vesicle size (nm). Table 1 lists the results of experiments with predicted responses by the mathematical model and those actually observed. The optimized SLNs (F-O) showed DEE of 51.55%, and vesicle size (nm) of 42.33% with small error values (4.81 and 4.20, respectively).

#### 3.1.2 FTIR

**Fig. 3:**
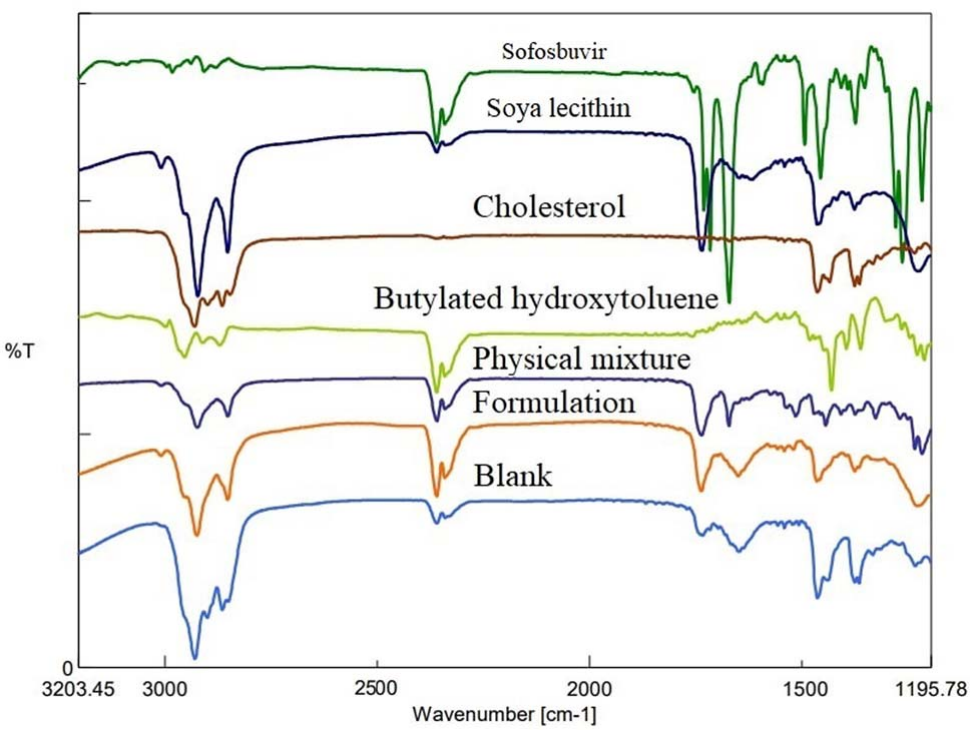
FTIR spectroscopic analysis of sofosbuvir, soy-lecithin (SL), cholesterol (CHL), butylated hydroxyl toluene (BHT), physical mixture, formulation (drug loaded) and blank formulation (without drug).

FTIR study depicted the absence of any strong physical interaction between the drug (sofosbuvir) and selected lipids/excipients. Characteristic peaks of sofosbuvir were observed at 3353.60 cm^−1^ (N–H stretching), 2978.52 cm^−1^ (C–H stretching), and 1721.16 cm^−1^ (C=O stretching). In the physical mixture of drug (sofosbuvir) and excipients, the characteristic peaks of sofosbuvir (*e.g.*, 2923.56 cm^−1^ related to C–H stretching and 3360.35 cm^−1^ related to N–H stretching) were also detected, although some minor shifting in the peak intensity was noticed. Similarly, characteristic peaks were noticed at 1514.81 cm^−1^ (N–O stretching) representing SL, 1735.62 cm^−1^ (C=O stretching) representing CHL and at 2953.45 cm^−1^ (C–H stretching) representing BHT (Fig. 3). The FTIR spectra of the physical mixture clearly showed no new peaks nor any major shifting in peak intensity as compared to the individual component peaks. The results thus overall confirmed the lack of any substantial interaction between the drug and excipients.

#### 3.1.3 DSC

**Fig. 4:**
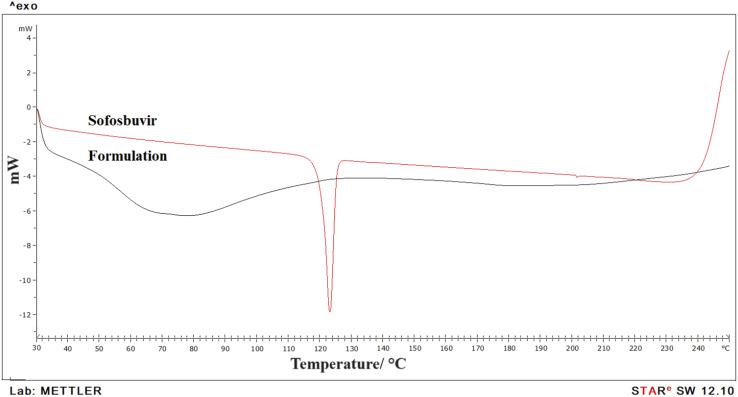
DSC analysis of pure sofosbuvir and optimized formulation, SLNs (F-O).

DSC was used to evaluate the physiochemical state of the medication and chemical interaction in the formulation (Fig. 4). Data showed a clear sharp single endothermic peak for pure sofosbuvir at 127.58 °C, corresponding to its melting point. However, in the optimized SLNs (F-O), no sharp peak was observed, which thus signified the loss of the crystalline property of the drug when encapsulated inside the LNs core. Further, no new endothermic peaks were detected in the optimized SLNs (F-O), even in the molten state, which also confirmed the absence of any chemical interaction and suitability of drug with excipients for formulation development.

#### 3.1.4 XRD

**Fig. 5:**
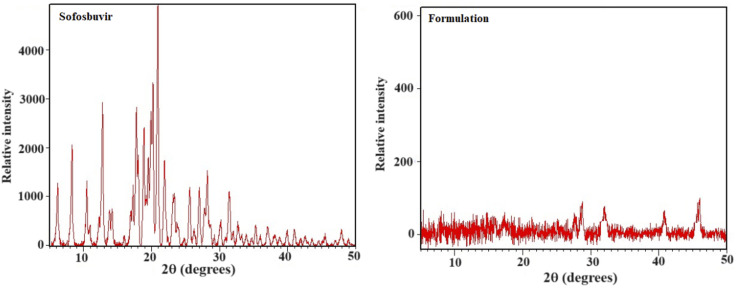
XRD analysis of pure sofosbuvir and optimized formulation, SLNs (F-O).

To get an idea on the crystalline/amorphous properties of sofosbuvir and the optimized SLNs (F-O), X-ray diffraction experiments were conducted. The crystalline nature of sofosbuvir was clearly indicated from the sharp diffraction peaks with high intensity at 10.504°, 10.975°, 12.307°, 12.822°, 17.278°, 17.766°, 20.225°, 25.583°, 27.767° and 45.398°. However, in the XRD spectrum of optimized SLNs (F-O), relatively weak intensity peaks were observed (Fig. 5). The decrease in the peak height/intensity in the optimized SLNs (F-O) signified the reduced crystallinity and the appearance of slight amorphization in the sample.

#### 3.1.5 Yield%, drug loading percentage (*in vitro*) and loading efficiency%

The yield% of optimized SLNs (F-O) was found to be 68.2%. The practical drug loading % of the optimized SLNs (F-O) was 8.31 ± 0.33% with drug loading efficiency of 51.55 ± 1.4% (Table 3). The reasonable practical drug loading capacity and good % yield of the selected formulation can be attributed to the optimized composition/process variables selected in the study.

**Table 3:** % yield, % drug loading and % drug efficiency of optimized formulation, SLNs (F-O)

Formulation code	Yield, %	Drug loading, %	Loading efficiency, %
Optimized SLNs (F-O)	68.2%	7.31 ± 0.33%	51.55 ± 1.4%

#### 3.1.6 Average vesicle size, PDI, and zeta potential measurement

**Fig. 6:**
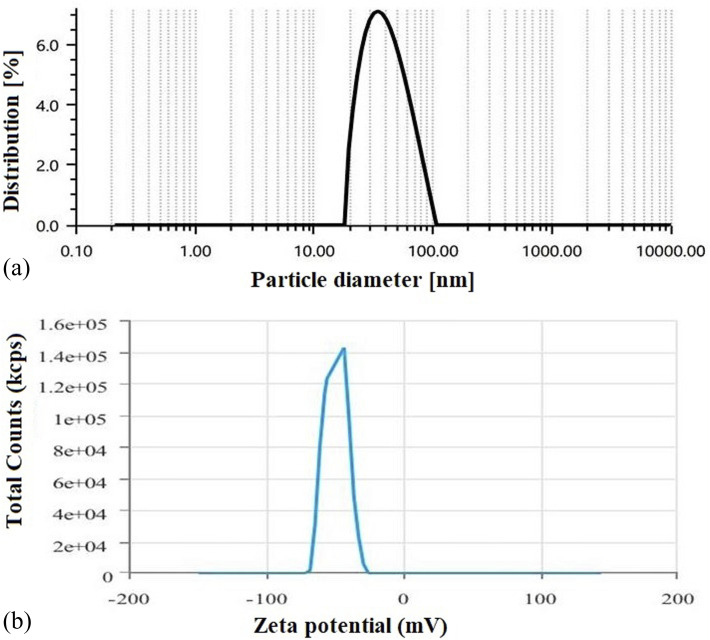
(a) Particle size analysis of optimized formulation, SLNs (F-O); (b) zeta potential determination of optimized formulation, SLNs (F-O).

DLS data showed that the optimized SLNs (F-O) were within the desired nano-sized range (Fig. 6a). The average size of the optimized SLNs (F-O) was found to be 42.43 nm with a PDI value of 0.53. The size data was also found to be closely related to the data predicted by factorial design. The zeta potential of the optimized SLNs (F-O) was found to be −49.89 mV (Fig. 6b). Thus, the optimized formulation showed a homogenous size distribution pattern, as depicted from its lower PDI value. The high negative surface potential of the optimized SLNs (F-O) suggests their better stability at the suspension stage.

#### 3.1.7 FESEM

**Fig. 7:**
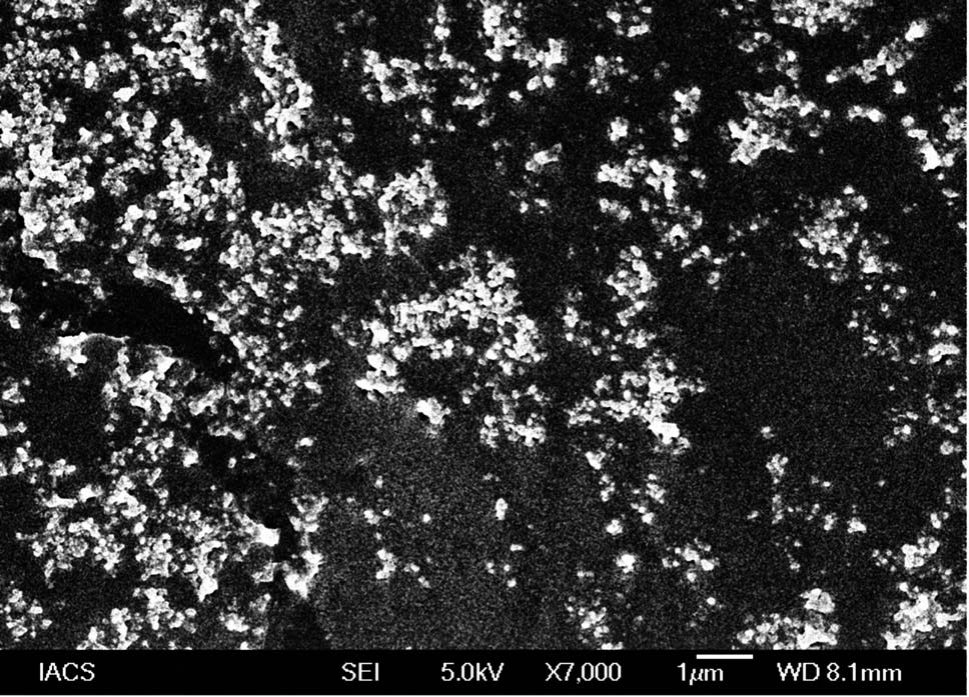
Field emission scanning electron microscopy of optimized formulation, SLNs (F-O).

The particle shape and external morphology of the optimized SLNs (F-O) were examined using FESEM (Model-JEISS, Japan). The optimized SLNs (F-O) showed a nano-size spherical structure with a smooth surface (Fig. 7). Although some agglomeration was visualized in the formulation, mostly a homogeneous distribution pattern was observed throughout the image. The vesicles were spherical shaped as shown in the FESEM image. Further, there were no indications of lumps or the production of larger agglomerates across the formulation, supporting the good formulation qualities.

### 3.2 *In vitro* drug release study (dialysis method)

**Fig. 8:**
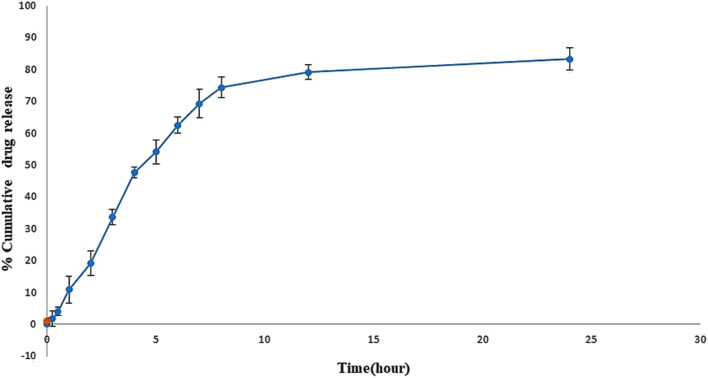
*In vitro* drug release study of optimized formulation, SLNs (F-O) in phosphate buffer saline, ph 7.4.

*In vitro* release study showed the sustained release of sofosbuvir from the optimized SLNs (F-O) (Fig. 8). Although the drug was released relatively faster in the initial hours of the study, which was obvious in most cases, with the passage of time, slower release of the sofosbuvir was observed. Within the reported time period of 24 h, a cumulative 83.29% of sofosbuvir was released from the optimized SLNs (F-O). The sustained sofosbuvir release from the optimized SLNs (F-O) favours their *in vivo* application.

#### 3.2.1 Estimation of drug release kinetics

To depict the mechanism of sofosbuvir release from the optimized formulation (F-O SLNs), the release data was fitted into various kinetic models. From the analysis of the corresponding *R*^2^ values of the graphs, the release was found to fit the Higuchi kinetic model well, with the best linearity and an *R*^2^ of 0.9482 (Table 4).

**Table 4:** Estimation of drug release kinetics

Sl. No.	Release kinetics model	The correlation coefficient of optimized SLNs (F-O)
1	Zero-order model	0.8242
2	First-order model	0.0265
3	Higuchi model	0.9443
4	Hixon–Crowell model	0.5622
5	Korsmeyer peppas model	0.5303

### 3.3 *In vivo* pharmacokinetic studies

#### 3.3.1 Plasma/brain PK study

**Fig. 9:**
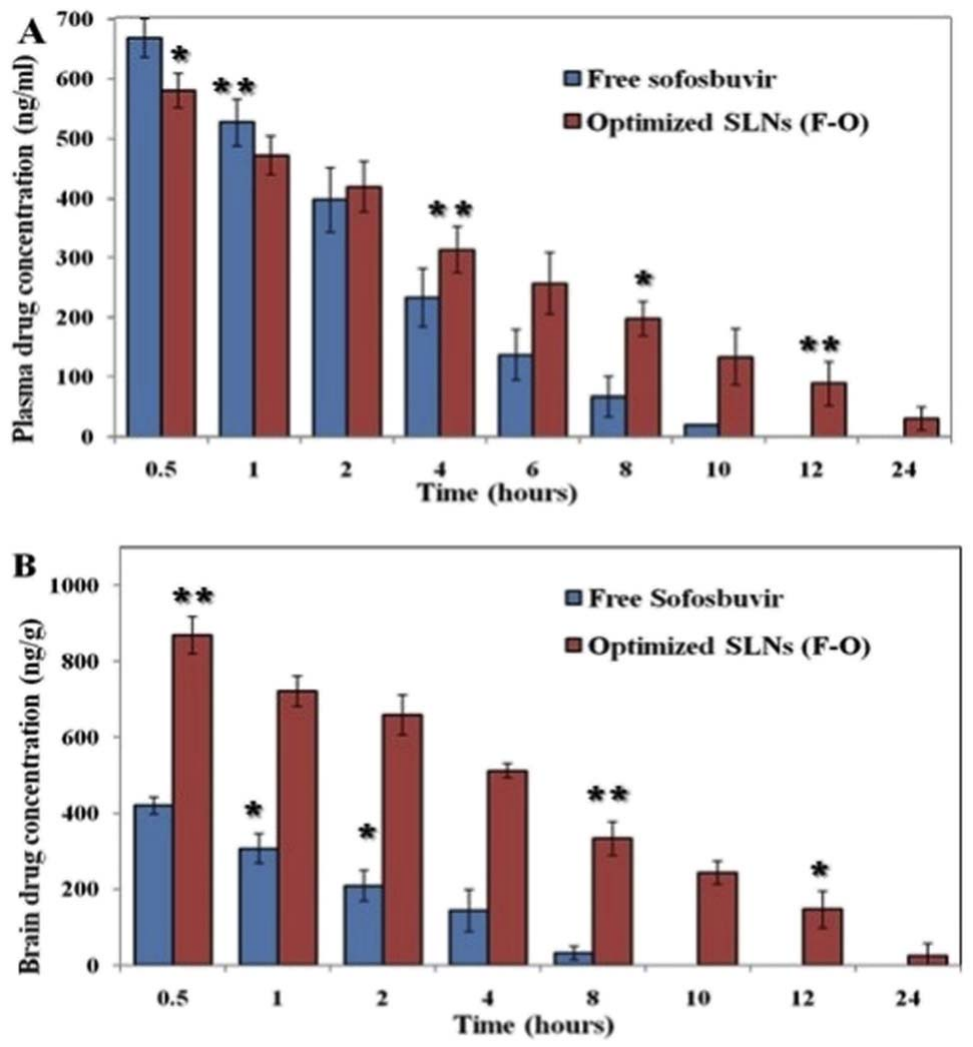
(A) Plasma pharmacokinetic analysis of drug after i.v. bolus administration of free sofosbuvir and SLNs (F-O) in Sprague-Dawley rats. (B) Brain pharmacokinetic analysis of drug after i.v. bolus administration of free sofosbuvir and SLNs (F-O) in Sprague-Dawley rats (**P < 0.01, 91 < 0.05).

The plasma PK study was carried out to determine the concentration of sofosbuvir in the blood of the experimental rats after administration of the optimized SLNs (F-O)/pure sofosbuvir at pre-fixed time intervals. The plasma PK data demonstrated a significant difference in the most important PK parameters between optimized SLNs (F-O) and free sofosbuvir treatment (Table 5). The plasma drug concentration *vs.* time graph clearly illustrates that the optimized SLNs (F-O) had a longer blood residence period than that of free sofosbuvir (Fig. 9A). The AUC_0–∞_ was found to be 15 372 ± 1233.5 ng h ml^−1^ for the optimized SLNs (F-O), whereas it was 6949.7 ± 225.2 ng h ml^−1^ for pure sofosbuvir. A higher AUC signifies higher bioavailability. Similarly, the MRT (15.4 h) and *V*_d_ (0.06 L) of the optimized SLNs (F-O) were also found to be improved significantly as compared to those for free drug administration (5.9 h and 0.02 L). MRT was improved almost three-fold for the optimized SLNs (F-O)-treated group compared to the free drug treatment group. At 24 h, the concentration of sofosbuvir was found to be 28.71 ± 9.22 ng ml^−1^ for the optimized SLNs (F-O), whereas it was non-detectable for the free sofosbuvir at the same time, confirming the sustained release of the drug from the optimized SLNs (F-O).

**Table 5:** Estimation of plasma and brain pharmacokinetic parameters in Sprague-Dawley rats after intravenous bolus administration of free sofosbuvir and optimized SLNs (F-O) suspension

PK parameter	Plasma^a^		Brain^a^	
	Optimized SLNs (F-O)	Free sofosbuvir	Optimized SLNs (F-O)	Free sofosbuvir
AUC _0–∞_ (ng h ml^−1^)	15 372 ± 1233.5	6949.7 ± 225.2*	10 523.6 ± 517.2*	3141.4 ± 255.7
AUMC_0–∞_ (ng h^2^ m^−1^)	81 345 ± 438.6	30 265 ± 2521.8	128 76.8 ± 1895.1	4418 ± 247.9*
MRT _0–∞_ (h)	15.4 ± 0.51	5.9 ± 0.8*	11.2 ± 5.1*	3.7 ± 0.11
CI (L h^−1^)	0.05 ± 0.1	0.81 ± 0.09*	0.07 ± 0.01	0.36 ± 0.44
*V*_d_ (L)	0.06 ± 0.01*	0.02 ± 0.003	0.38 ± 0.07	0.05 ± 0.31*

a Data show mean ± SD (*n* = 6). AUC: area under the plasma concentration–time curve; AUMC: area under the first moment curve; Cl: clearance; MRT: mean residence time; *V*_d_: steady state volume of distribution. * Data were significantly different (*p* < 0.05) where free sofosbuvir and optimized SLNs (F-O) were compared. It was assessed by one-way analysis of variance (ANOVA) through Tukey–Kramer's multiple comparisons test.

Brain PK data also showed a similar trend to the plasma PK data in the experimental animal groups (Fig. 9B). The calculated AUC_0–∞_ of the optimized SLNs (F-O)-treated group was found to be 10 523.6 ± 517.2 ng h ml^−1^, whereas the same was 3141.4 ± 255.7 ng h ml^−1^ for the free sofosbuvir-treated group. Substantial differences were also found between the optimized SLNs (F-O) and the free sofosbuvir-treated groups in terms of the AUMC_0–∞_, *V*_d_, and MRT values. A higher MRT was observed for the optimized SLNs (F-O) (11.2 h) than the free sofosbuvir (3.7 h) (Table 5). Moreover, the optimized SLNs (F-O) had a higher volume of distribution (0.38 ± 0.07 L) with a lower rate of clearance (0.07 ± 0.01 L h^−1^) than those of the free drug. Higher AUC, MRT, *V*_d_ and lower clearance rate of the optimized SLNs (F-O) signified its higher brain availability than that of free sofosbuvir.

#### 3.3.2 *Ex vivo* hemolysis study

**Fig. 10:**
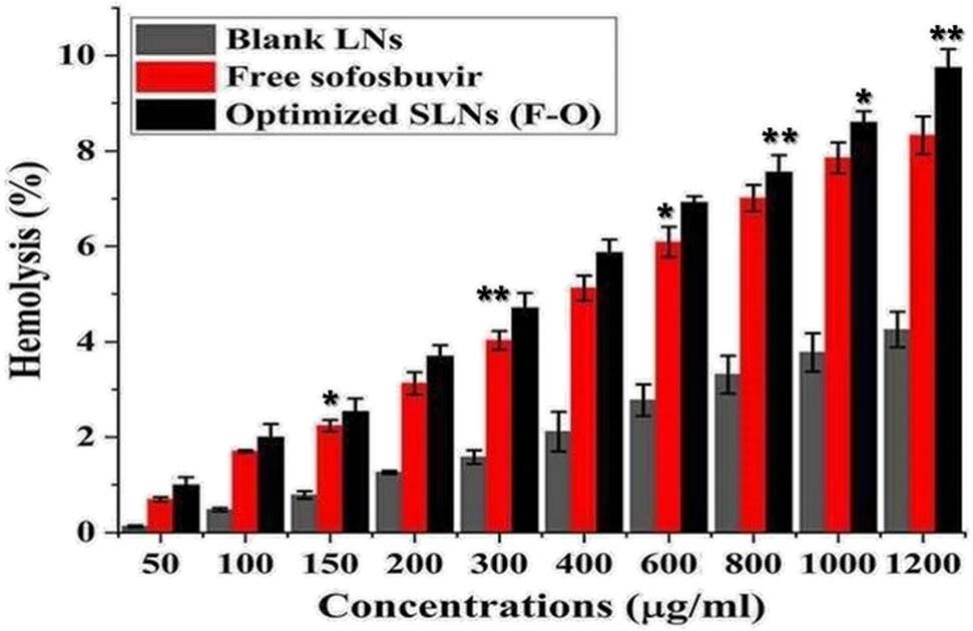
Hemolysis% assay of free sofosbuvir, blank LNLs (without drug) and optimized SLNs (F-O) in human red blood cells. (**p < 0.01, *p < 0.05).

Hemolytic assays were performed on rat RBCs *ex vivo* to determine the blood compatibility of the optimized SLNs (F-O) and sofosbuvir-free LNs in combination with free sofosbuvir at various concentrations (50–1200 μg ml^−1^) (Fig. 10). As observed from the study, negligible cytotoxicity was observed for the optimized SLNs (F-O) and free sofosbuvir. Although the hemolysis caused by optimized SLNs (F-O)/free sofosbuvir was more than that in the blank LNs-treated group, the overall hemolysis percentage was below 9%. Even at the highest tested concentrations, the toxic effect was low, confirming the non-toxic nature of the formulation (drug loaded/blank). Overall, the hemolytic effect was in the order of blank LNs < free sofosbuvir < optimized SLNs (F-O). The lower hemolytic effect of the optimized SLNs (F-O) signifies its biocompatible nature.

### 3.4 *In silico* docking analysis

**Fig. 11:**
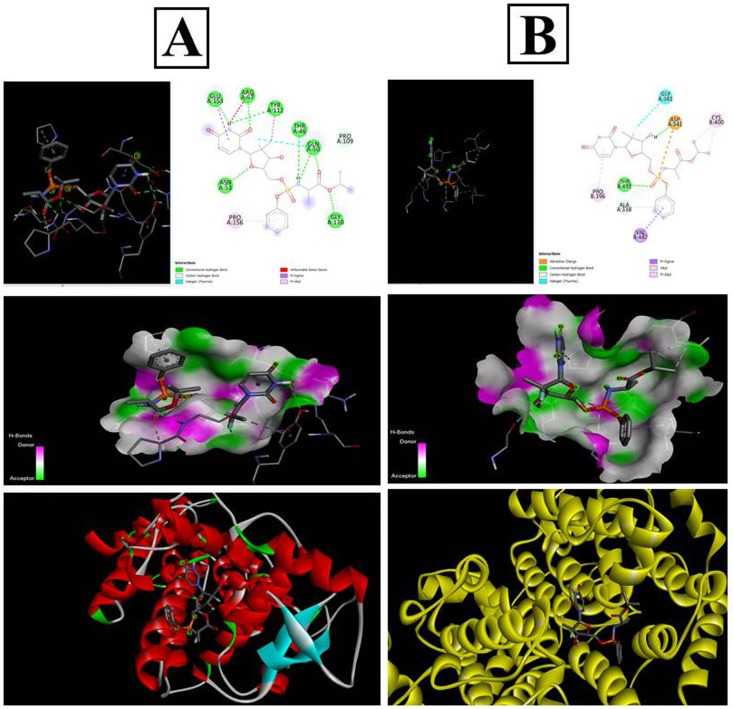
*In silico* docking analysis of sofosbuvir with the (A) 4YXP (Herpes simplex protein) and (B) 7EWQ (mumps viral protein). Analysis was conducted using AutoDockTools-1.5.6 and PyRx software.

The PubChem database was used to depict the 2D structures of all components of sofosbuvir and the selected proteins (Fig. 11). The top proteins from herpes simplex and mumps viruses were docked with sofosbuvir. Thus, two sets of docking results are shown. Data showed that the tested herpes simplex virus protein (4YXP) has a strong ability to bind with the selected ligand (sofosbuvir) with a docking score of −7.5. Similarly, reasonable binding ability of the drug was observed against mumps viral protein (7EWQ) with docking score of −7.3. In view of the successful ligand–receptor interaction, sofosbuvir has potential to be used against herpes simplex- and mumps-related viral meningitis.

## 4. Discussion

Transport of hydrophilic therapeutic molecules to the brain through lipophilic nanovesicular carriers has been a recent trend in drug delivery research. Sofosbuvir, a BCS class III drug owing to its hydrophilicity, has a limited role in brain disorders. Thus, delivery of sofosbuvir to rat brain tissue through optimized SLNs (F-O) has been attempted in the work. Various formulations were developed by applying a 2^3^ factorial design approach. Three major formulation factors, *viz*. amount of SL, amount of CHL and speed of centrifugation, were varied and their corresponding effects on the responses of drug encapsulation efficiency (%) and average vesicle size (nm) were noted. Out of several batches prepared based on factorial design data, F-O (prepared using 100 mg of SL, 60 mg of CHL, and 10 000 speed of centrifugation) was selected as the optimized formulation with DEE of 51.55%, and vesicle size (nm) of 42.33% with small error values (4.81 and 4.20, respectively). From the 2D contour plots and 3D response surface plots relating to the responses analysed in the present work (drug encapsulation efficiency and average vesicle size), both these responses increased with the increment of the amounts of SL and CHL used to prepare the SLNs. However, no marked effect of centrifugation speed on both the responses was noticed.

FTIR spectroscopy is used to analyse the interaction of infrared radiation with matter, which can offer important information on molecular structure and interaction of the test material(s). In our case, FTIR data revealed no significant interaction between the drug and the selected lipids/excipients. However, there was a minor physical interaction as some minor shifting in the characteristic peaks was observed from the individual components to the physical mixture/formulation. Such physical interactions play a key role in the successful formation of the vesicular nanostructures. However, the absence of any major shifting in the characteristic peaks of drug/excipient justified well compatibility of sofosbuvir with selected lipids/excipients.

DSC data showed a single sharp endothermic peak for pure sofosbuvir, which was absent in the case of the formulation. It thus indicated successful encapsulation of the drug within nanovesicular structures with reduced crystallinity. Usually, the presence of a sharp melting endotherm signifies crystalline property, whereas a flattened or broader peak without sharpness signifies amorphous nature. Clearly, it was observed that the drug was encapsulated inside the vesicular core with loss of crystallinity. Similar observations in DSC thermograms regarding decreased crystallinity of drugs encapsulated in nanoformulations were also reported by other researchers.^26^ Further, there was no chemical interaction between sofosbuvir and the selected lipids as no shifting in endothermic peak of drug or appearance of new peaks were detected in the optimized SLNs (F-O).

XRD is a useful tool for identifying the crystalline phases, degree of crystallinity, and texture of polycrystalline samples.^27^ In the optimized SLNs (F-O), the XRD peaks were not as sharp as the peaks for pure sofosbuvir, which was another clear indication of the slightly amorphous nature of the formulation. Although no such new peaks were detected in the optimized SLNs (F-O), a change in peak height/intensity was observed as compared to the XRD diffractogram for pure sofosbuvir. However, the decreased crystallinity of the drug would not alter its action, rather it would be helpful for improvement of the dissolution profile of the drug encapsulated in the vesicular lipid core. Similar findings related to XRD data were also reported in other studies, where encapsulation of a drug in a nanocarrier core led to decreased sharpness or width/height of the peaks (amorphization).^28,29^ However, in those studies, there were no reports were related of changes in drug efficacy following amorphization.

FESEM analysis depicted the formation of spherical shaped vesicles within the nano-size range (less than 100 nm) with a smooth surface. The vesicles were mostly homogenous in size range. However, the overall size of the vesicles was found to be in good agreement with the DLS data. Despite the close proximity of the vesicles, there were no signs of bigger lumps in the sample, justifying the suitability of standardized process parameters used for formulation development.

Like FESEM, the PDI is also a significant indicator of the stability, size distribution and homogeneity of a colloidal dispersion system. A low PDI suggests more homogeneous distribution of colloidal vesicles with higher stability. In contrast, a higher PDI score indicates particle aggregation with low suspension stability. The optimized formulation (F-O SLNs) exhibited a PDI of 0.53, which thus suggests that the formed vesicles were relatively uniform in size and spread uniformly throughout the formulation. Zeta potential is another key parameter in predicting the stability of colloidal particles in a dispersion, as it determines the electrostatic repulsion/attraction between particles.^22^ A zeta potential greater than +30 mV or less than −30 mV usually indicates that particles are stable in colloidal dispersion.^14^ In our study, the optimized SLNs (F-O) showed a high negative zeta potential (−49.2 mV). This indicates that the formulation would have greater physical stability in suspension form.

The purpose of an *in vitro* release study is to determine how quickly and to what extent the drug is released from the delivery system under simulated physiological conditions. During the release study period, the optimized SLNs (F-O) released 83.29% of the sofosbuvir. Although initially higher release of sofosbuvir was observed from optimized SLNs (F-O), the overall release pattern clearly depicted sustained release. Further, when the release data was fitted into various kinetic models, the Higuchi model showed the best linearity (*R*^2^ = 0.9482), which indicates a non-Fickian drug diffusion pattern and suggests that the release of the sofosbuvir from the vesicular lipid core might follow a diffusion and erosion mechanism.

PK describes the movement pattern of a therapeutic molecule throughout the body, which can be overall stated the actions of the body on the therapeutic molecule. For drugs to be approved by regulatory bodies before clinical use, PK data is crucial. Important PK parameters like AUC, MRT, and *V*_d_ have significant meaning in relation to the therapeutic effectiveness and bioavailability of drugs. The area under a plot of drug plasma concentration *versus* time following administration of a drug is normally referred to as ‘area under the curve’ (AUC). AUC depicts the extent of exposure of a drug inside the body and its rate of clearance. Overall, a higher AUC signifies higher bioavailability. Mean residence time (MRT) is another important PK parameter, which is considered crucial for drugs that are administered intravenously (i.v. bolus/infusion) in a one- or two-compartment system following Michaelis–Menten elimination. Overall, MRT represents the average duration of residence of a drug in the body. Similarly, volume of distribution (*V*_d_), another important PK parameter, serves as an indicator of the extent to which a drug is distributed from plasma throughout the tissues. It is used to estimate the dose of drug required to achieve a steady-state plasma concentration. The higher the *V*_d_, the higher the distribution of the drug in other tissues (extravascular compartment). In turn, *V*_d_ is a prime determinant of the half-life of a drug.

Our *in vivo* PK study clearly depicted improved plasma/brain PK parameters in animals treated with optimized SLNs (F-O) as compared to those treated with free sofosbuvir post *i.v.* bolus. The plasma drug concentration in the optimized SLNs (F-O)-treated group was higher than that in the free sofosbuvir-treated group after 24 hours. The drug concentration was undetectable in the free drug-treated groups at 24 h, whereas it was 28.71 ng ml^−1^ in the optimized SLNs (F-O)-treated group. A clear difference in MRT was also observed between the two groups with an almost three-fold increase for the optimized SLNs (F-O)-treated group. Other important parameters, such as AUMC, *V*_d_, and Cl, were also found to be significantly different in the optimized SLNs (F-O) and free sofosbuvir-treated groups, justifying the higher bioavailability and longer residence time of the optimized SLNs (F-O) compared to the free sofosbuvir. A similar observation was noted for the brain PK study. The MRT of the optimized SLNs (F-O) in the brain was increased by more than 3.5 times compared to the MRT of free sofosbuvir. Even at 10 h, the concentration of sofosbuvir was no longer detectable in the free drug-treated group, since it had gone beyond the threshold detection limit of the LC-MS/MS method. However, from the optimized SLNs (F-O) sofosbuvir was detected even at 24 h. Clearly, the data showed that the optimized SLNs (F-O) demonstrated significantly prolonged accumulation in brain tissue. In view of the desired nano-size, highly lipophilic nature and higher negative surface charge, the optimized SLNs (F-O) might successfully bypass the trap of reticuloendothelial cells and could remain in the brain for sufficient period of time.

As blood compatibility is an important criteria for formulations intended for parenteral application, *ex vivo* hemolysis assay has become a widely accepted method for validating the *in vivo* application of novel parenteral products. The study depicted negligible hemolysis in rat RBCs treated with the optimized SLNs (F-O)/blank LNs. Thus, the formulation was proved to be blood-compatible, non-toxic and could be safely used for further *in vivo* studies.

*In silico* docking study of sofosbuvir with 4YXP (herpes simplex protein) and 7EWQ (mumps viral protein) showed effective binding of the selected proteins with sofosbuvir. The lower the binding energy, the tighter the bond would be. Thus, lower docking score signifies higher binding interaction. In this study, a reasonable docking score of −7.5 for sofosbuvir and 4YXP and −7.3 for sofosbuvir and 7EWQ showed satisfactory molecular interaction of the drug and ligands. The study thus provided desirable information on the rationality of use of sofosbuvir in viral meningitis.

## 5. Conclusion

This work attempted to improve the brain delivery of a potent water-soluble antiviral drug *via* an experimentally developed lipid nanocarrier as a futuristic strategy for effective treatment of meningitis. SLNs were successfully developed using 2^3^ factorial design and the results were well correlated with actual data. FTIR/DSC studies showed the compatibility of sofosbuvir with the excipients. The optimized SLNs (F-O) had spherical shape, smooth surface, desirable nano-size, low PDI, and 8.31% loading capacity with sustained release of sofosbuvir *in vitro*. The optimized SLNs (F-O) exhibited longer MRT and AUC than those of free sofosbuvir, as assessed from PK data. Hemolysis data showed the blood-compatible nature of the optimized SLNs (F-O). Satisfactory molecular interaction of sofosbuvir with herpes simplex and mumps viral proteins provided rationale for the potential application of sofosbuvir in viral meningitis. Further studies are under way to check the *in vivo* effectiveness of optimized SLNs (F-O) in animal models.

## Author contributions

Bhabani Sankar Satapathy: conceptualization, draft preparation, data analysis. Pralaya Kumar Sahoo: literature search, material preparation. Snigdha Pattnaik: original draft preparation and editing. Amit Kumar Nayak: critically revised the work. Laxmidhar Maharana: data analysis. Rudra Narayan Sahoo: references arrangement and editing.

## Conflicts of interest

The authors of the article have no conflicts of interest to declare.

## Supplementary Material

## References

[cit1] Zhang W., Mehta A., Tong Z., Esser L., Voelcker N. H. (2021). Advanced Science.

[cit2] Khaledian S., Dayani M., Fatahian A., Fatahian R., Martinez F. (2022). J. Mol. Liq..

[cit3] Khatoon R., Alam M. A., Sharma P. K. (2021). J. Drug Delivery Sci. Technol..

[cit4] Javed S., Mangla B., Almoshari Y., Sultan M. H., Ahsan W. (2022). Nanotechnol. Rev..

[cit5] Kaur H., Kumar V., Kumar K., Rathor S., Kumari P., Singh J. (2016). Curr. Pharm. Des..

[cit6] Aguilar-Pérez K. M., Avilés-Castrillo J. I., Medina D. I., Parra-Saldivar R., Iqbal H. M. (2020). Front. Bioeng. Biotechnol..

[cit7] Dahiya M., Dureja H. (2018). Curr. Nanomater..

[cit8] Soema P. C., Willems G. J., Jiskoot W., Amorij J. P., Kersten G. F. (2015). Eur. J. Pharm. Biopharm..

[cit9] Mukherjee B., Mondal L., Chakraborty S., Paul P., Choudhury A., Bhattacharya S., Hossain M. C. (2015). Curr. Pharm. Biotechnol..

[cit10] Chugh Y., Dhiman R. K., Premkumar M., Prinja S., Singh Grover G., Bahuguna P. (2019). PLoS One.

[cit11] El-Shafai N. M., Masoud M. S., Ibrahim M. M., Ramadan M. S., Mersal G. A. M., El-Mehasseb I. M. (2022). Int. J. Biol. Macromol..

[cit12] Awan A. N., Khalid R., Javed A., Shah M. R., Ali S. A. (2023). Plasmonics.

[cit13] Mesci P. , Macia A., Saleh A., Martin-Sancho L., Yin X., Snethlage C., Avansini S., Chanda S. K., and Muotri A., bioRxiv, 2020, preprint, pp. 2020–2105, arXiv:2020.05.30.125856, 10.1101/2020.05.30.125856

[cit14] Satapathy B. S., Mukherjee B., Baishya R., Debnath M. C., Dey N. S., Maji R. (2016). RSC Adv..

[cit15] Alavi M., Rai M., Varma R. S., Hamidi M., Mozafari M. R. (2022). Micro Nano Bio Asp..

[cit16] Pramod K., Suneesh C. V., Shanavas S., Ansari S. H., Ali J. (2015). J. Anal. Sci. Technol..

[cit17] Leyva-Porras C., Cruz-Alcantar P., Espinosa-Solís V., Martínez-Guerra E., Piñón-Balderrama C. I., Compean Martínez I., Saavedra-Leos M. Z. (2019). Polymers.

[cit18] Bunaciu A. A., UdriŞTioiu E. G., Aboul-Enein H. Y. (2015). Crit. Rev. Anal. Chem..

[cit19] Bhatt P., Lalani R., Vhora I., Patil S., Amrutiya J., Misra A., Mashru R. (2018). Int. J. Pharm..

[cit20] Karmakar S. A. (2019). Recent Trends Mater. Phys. Chem..

[cit21] Zheng Y., Cosgrove D. J., Ning G. (2017). Microsc. Microanal..

[cit22] Satapathy B. S., Kumar L. A., Pattnaik G., Barik B. (2021). Acta Chim. Slov..

[cit23] Mondal L., Mukherjee B., Das K., Bhattacharya S., Dutta D., Chakraborty S., Pal M. M., Gaonkar R. H., Debnath M. C. (2019). Int. J. Nanomed..

[cit24] Panda J., Satapathy B. S., Mandal B., Sen R., Mukherjee B., Sarkar R., Tudu B. (2021). J. Microencapsulation.

[cit25] Chen W., Kan H., Qin M., Yang J., Tao W. (2023). BioMed Res. Int..

[cit26] Ang C. W., Tan L., Qu Z., West N. P., Cooper M. A., Popat A., Blaskovich M. A. (2021). ACS Biomater. Sci. Eng..

[cit27] Satapathy B. S., Patel A., Sahoo R. N., Mallick S. (2021). J. Serb. Chem. Soc..

[cit28] Gou K., Wang Y., Guo X., Wang Y., Bian Y., Zhao H., Guo Y., Pang Y., Xie L., Li S., Li H. (2021). Acta Biomater..

[cit29] Ang C. W., Tan L., Qu Z., West N. P., Cooper M. A., Popat A., Blaskovich M. A. (2021). ACS Biomater. Sci. Eng..

